# Construction of a Full-Atomic Mechanistic Model of Human Apurinic/Apyrimidinic Endonuclease APE1 for Virtual Screening of Novel Inhibitors 

**Published:** 2012

**Authors:** I.G. Khaliullin, D.K. Nilov, I.V. Shapovalova, V.K. Švedas

**Affiliations:** Belozersky Institute of Physicochemical Biology, Lomonosov Moscow State University; Lomonosov Moscow State University, Faculty of Bioengineering and Bioinformatics

**Keywords:** apurinic/apyrimidinic endonuclease, QM/MM, enzymatic mechanism, molecular modeling, inhibition

## Abstract

A full-atomic molecular model of human apurinic/apyrimidinic endonuclease APE1, an important enzyme in the DNA repair system, has been constructed. The research consisted of hybrid quantum mechanics/molecular mechanics modeling of the enzyme-substrate interactions, as well as calculations of the ionization states of the amino acid residues of the active site of the enzyme. The choice of the APE1 mechanism with an Asp210 residue as a proton acceptor was validated by means of a generalization of modeling and experimental data. Interactions were revealed in the active site that are of greatest significance for binding the substrate and potential APE1 inhibitors (potential co-drugs of interest in the chemo- and radiotherapy of oncological diseases).

## INTRODUCTION 

DNA damages occur frequently as a result of replication errors or on exposure to various exo- and endogenic factors, such as ultraviolet radiation and oxidative stress. In order to ensure genomic stability, mammals possess enzymatic repair systems – direct repair, base/nucleotide excision repair, and recombination mechanisms – which facilitate the elimination of most forms of DNA damage [1–3]. The pharmacological inhibition of the repair systems is a promising method for improving the efficacy of oncological therapy. The reason which accounts for this fact is that the repair systems resist the effect of the chemotherapeutic agents (e.g., temozolomide and cisplatin [4]) which damage the DNA in order to kill the tumor cell. Therefore, the selective inhibition of the enzymes participating in the DNA repair processes can be used as an accompanying therapy. The agents whose binding in the enzyme active site affects the residues directly participating in the catalytic mechanism are reasonably expected to exhibit the highest levels of efficacy. Therefore, adequate data regarding the organization of the active site of the target enzyme, the charge distribution, and analysis of the interactions determining the strength of substrate–inhibitor binding are fundamentally necessary in the search for novel potential pharmaceuticals in the field of cancer therapy. 

Apurinic/apyrimidinic endonuclease 1 (APE1) is the key enzyme in the DNA repair system, known as “base excision repair” (BER). Apurinic/apyrimidinic (AP) sites are the deoxyribose residues in the DNA molecule without a nitrogenous base; they result from the enzymatic hydrolysis of the N-glycoside bond of a damaged nucleotide and actually are the intermediates of the BER process. Furthermore, the AP sites may spontaneously emerge in cells due to apurinization [5]. According to current estimations, up to 10,000 AP sites are formed in mammalian cells per day [6]. The APE1 endonuclease recognizes the AP sites and hydrolyzes their 5’-phosphodiester bond for subsequent replacement with an undamaged nucleotide [5, 7]. The data obtained in laboratory and clinical studies attest to the significant role played by this enzyme in the development of a tumor and in the appearance of tumour resistance to antitumor agents [8]. 

**Fig. 1:**
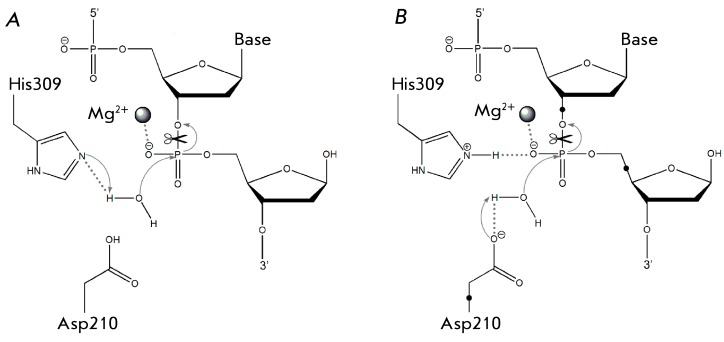
Basic concepts of APE1 catalytic mechanism found in literature: A) the His309 residue activates a water molecule acting as a base; B) the Asp210 residue activates a water molecule acting as a base, while the His309 residue participates in substrate binding. The link atoms used in the present work on QM/MM modeling of APE1 are depicted as ●.

There exists a wide variety of viewpoints concerning the catalytic mechanism underlying the action of endonuclease APE1. The first crystal structure of the enzyme was obtained in 1997 (PDB ID 1bix) [9]. In their description, the authors proposed a mechanism in which the role of the general base in the catalysis was attributed to the His309 residue. In this hypothetical mechanism, the uncharged residue His309, jointly with Asp283, forms a proton relay system similar to the one formed by serine proteases. The only difference is that a water molecule acts as an activated nucleophilic agent ( *Fig. 1A* ). The role of a metal ion in this mechanism consists in the binding and polarization of the negatively charged phosphate group of the substrate and in the stabilization of the transition state of the enzymatic reaction. 

The fundamental significance of another residue in the active site (Asp210) for the catalysis was demonstrated in studies performed using site-directed mutagenesis: mutant forms of the enzyme with the substitutions Asp210Ala and Asp210Asn almost completely lost their catalytic properties (more than 25,000-fold reduction in activity was observed) as compared to the wild-type enzyme [10]. The determination of the crystal structures of human APE1 in complex with DNA derivatives resulted in a major revision of the assumptions regarding the mechanism of action of the enzyme [11]. One of the ascertained structures (PDB ID 1de8) is a complex of an inactive enzyme containing no metal ions with a substrate analogue, whereas the second structure (PDB ID 1de9) contains a metal (bivalent manganese) ion and the enzyme-bound DNA analogue of the substrate after catalytic cleavage. The conception was made regarding the structure of the enzyme–substrate complex, which simultaneously contains both the substrate analogue and a metal ion via the combination (spatial superposition) of the structures. Although the resulting model structure of the enzyme–substrate complex does not contain water molecules potentially capable of attacking the substrate, the arrangement of the residues in the active site before and after the catalytic process allowed making assumptions regarding the alternative mechanism of the catalytic reaction [11]. In the scheme proposed, the Asp210 residue acts as a general base activating the water molecule, whereas the His309 residue, along with the metal ion, participates in the binding and coordination to the phosphate group of the substrate ( *Fig. 1B)* . It is assumed that the positive charge of the His309 residue participates in the catalytic process, which is presumably facilitated by the proximate location of the Asp238 residue. The authors [11] attribute the major stabilizing function in the formation of the transition state of the enzymatic reaction to the Asn212 residue. 

Hypotheses postulating that a secondary metal binding area exists in the enzyme active site have been put forward in subsequent crystallographic [12] and molecular dynamics (MD) [13] studies. The “two metal ions” mechanism of action of APE1 [12] (similar to that revealed in a related enzyme, endonuclease Endo IV) and the “moving metal” mechanism [13], involving the moving of the magnesium ion between two binding sites during the catalytic process, were proposed in these works. 

It should be noted however that the NMR study utilizing the ^25^ Mg isotope [14] did not confirm the hypothesis of secondary magnesium ion binding in the active site of endonuclease APE1, thereby casting doubt on the “two metal ions” and the “moving metal” mechanisms. The authors of study [14] attribute the results of crystallographic studies [12] to the artefacts caused by the use of the lead ion instead of the magnesium ion. In turn, the effect of the motion of the metal ion during the MD modeling can be caused by the inaccuracy and approximations of the classical MD method. 

Mundle et al. [15] proposed a two-step variant of the APE1 mechanism involving the Tyr171 residue acting in the form of the phenolate ion for a direct nucleophilic attack on the phosphate group of the substrate. This conclusion was drawn on the basis of the data obtained via site-directed mutagenesis on the 171 ^st^ position. The kinetic studies of the catalytic properties of the mutated forms Tyr171Ala, Tyr171Phe, and Tyr171His demonstrated a fall in enzymatic activity by almost five orders of magnitude. It should be noted however that the authors [16] subsequently admitted the inconsistency of the previously proposed two-step scheme and supported the one-step mechanism, in which the His309 residue acts as the general base activating the water molecule, while the Tyr171 residue participates in the binding and proper orientation of the substrate. 

The molecular modeling methods could assist considerably in the study of the mechanism of action of the enzyme; however, this approach has not been extensively explored in the study of APE1. Modeling of the inhibitor–enzyme interaction was performed with no allowance for the ionization state of the inhibitor, thereby making the interpretation of the obtained results more difficult [17].  

Thus, there exists no unambiguous conception regarding the mechanism of action of human endonuclease APE1 or the role of the amino acids of the active site in binding and catalysis. Therefore, shedding more light on the structure of the active site, the nature of the interactions between the enzyme and substrate or inhibitors and the involvement of the active site residues into the catalytic mechanism of APE1 appears to be a topical task in molecular modeling. 

## MATERIALS AND METHODS 

### Molecular modeling software  

The ionization states of the amino acid residues were calculated using the PROPKA 2.0 software [18, 19]. The preparation of the initial structure for simulations and the trajectory analysis were performed using the AmberTools 1.2 package (http://ambermd.org). The energy minimization and MD simulations were performed using the Amber 10 package [20, 21]; the molecular docking was performed using the Lead Finder 1.1.14 program (MolTech Ltd., Russia) [22]. Modeling of the spatial structure of 6-hydroxy-DOPA was performed using the ACD/ChemSketch 8.17 program [23]. The visualization of the structures and trajectories was carried out using the VMD 1.8.6 software [24]. Parallel computations of the molecular dynamic trajectories were run on the SKIF-MSU “Chebyshev” supercomputer (MSU Research Computing Center). 

### Structure preparation 

The initial model of the APE1 enzyme–substrate complex was built on the basis of the 1de8 crystallographic structure [11]. The coordinates of the attacking water molecule were calculated by docking. The coordinates of the manganese ion were transferred from the 1de9 structure; the metal type was subsequently substituted for magnesium. The structure of the enzyme–substrate complex was protonated and placed into a box of TIP3P type water molecules with the shortest distance of 12 Å between the box edge and protein. Sodium ions were added to neutralize the charge of the system. The *ff99SB* force field [25] was used to describe the protein and DNA molecules; parameters from the R.E.DD.B database (http://q4md-forcefieldtools.org) [26] were used to describe the AP site. 

### Energy minimization and molecular dynamics 

The model of the APE1 enzyme–substrate complex was equilibrated and subjected to calculation of the 1000 ps MD trajectory according to the following protocol. Initially, the two-stage energy minimization of the solvated system was performed. At the first stage (2,500 steps of the steepest descent algorithm followed by 2,500 steps of the conjugate gradient algorithm), the molecular mechanics description of the system was performed with the coordinates of the protein, DNA, and magnesium ion being fixed by position restraints * k* (Δ *x* ) ^2 ^ with a force constant of 2 kcal/(mol·Å ^2^ ). At the second stage (5,000 steepest-descent steps followed by 5,000 conjugate-gradient steps), the system was divided into a quantum mechanical (QM) region and a molecular mechanical (MM) region; energy minimization was performed without any restraints. The QM region included the side chain of the Asp210 residue of the active site, the attacking water molecule, and the AP site fragment; this region was described by the RM1 semi-empirical Hamiltonian [27]. The link atom model was used to make allowance for the bonds crossing the QM–MM boundaries. 

After energy minimization, using the aforementioned QM/MM division the system was heated from 0 to 300 K over 50 ps (with positional restraints of 1 kcal/(mol·Å ^2^ ) on the protein, DNA, and magnesium ion), equilibrated over 500 ps at 300 K, and finally simulated for 1,000 ps. All simulations were performed using periodic boundaries and the PME (Particle Mesh Ewald) method to allow for long-range electrostatic interactions. The cut-off radius of the non-bonded interactions was 10 Å. The system was heated at a constant volume; the equilibration and 1,000 ps trajectory simulation were performed under constant pressure. The temperature was controlled by the Langevin method. The integration time step was 0.002 ps. Interatomic distances and angles in the active site of APE1 were estimated by analyzing the 1,000 ps trajectory of equilibrium simulation. 

### Molecular docking 

**Table:** . Distance characteristics of the APE1 enzyme–substrate complex obtained via the equilibrium 1,000 ps QM/MM MD simulation. Mean values are presented together with the standard deviation

Interaction	Distance, Å
H_2_O:O···AP site:P	1.91 ± 0.03
H_2_O:O···His309:HE2	2.52 ± 0.17
H_2_O:H_1_···Asp210:OD1	1.49 ± 0.07
H_2_O:H_2_···Asn212:OD1	2.62 ± 0.47
AP site:O1P···Mg^2+^	1.84 ± 0.04
AP site:O1P···His309:HE2	1.78 ± 0.09
AP site:O2P···Asn212:HD2	2.08 ± 0.32
dC5:O3’···Mg^2+^	1.95 ± 0.06
Asp210:OD2···Asn212:H	2.22 ± 0.20

The model for performing the molecular docking procedure was obtained as follows. Water molecules, sodium ions, and the DNA substrate analogue molecule were removed from the structure of the solvated APE1 enzyme–substrate complex after energy minimization. The energy grid map surrounding the AP-site area was then calculated. Finally, a potential inhibitor molecule, 6-hydroxy-DOPA, was docked into the active site with the use of the genetic search algorithm implemented in the docking program. 

## RESULTS AND DISCUSSION 

### Ionization states of the active site residues 

Based on the results of the calculation of the ionization states of the active site residues using the PROPKA 2.0 method, it was determined that the His309 residue is protonated (the calculated *pK*_a _ value of 8.6 matches the *pK*_2 _ value of the experimentally determined pH profile of enzyme activity) under optimal conditions of the hydrolysis of the phosphodiester bond (pH 7–8 [12]), whereas the Asp210 residue is deprotonated (the calculated *pK*_a_ value of 6.2 is close to the pK1 value of the pH profile of enzyme activity). Thus, it can be said that the deprotonated and negatively charged Asp210 residue acts as a general base in catalysis, whereas the positively charged His309 residue participates in the binding of the negatively charged phosphate group of the substrate and in stabilization of the reaction intermediate product. Therefore, when building the full-atomic model of APE1, Asp210 and His309 were modelled in their charged forms. 

### Model of the enzyme-substrate complex and the deduced catalytic mechanism of action of APE1 endonuclease  

The starting solvated model of the enzyme–substrate complex APE1 was created on the basis of the 1de8 and 1de9 crystallographic structures as described in Materials and Methods. It was then necessary to optimize the atom positions within the model (especially the coordinates of the hydrogen atoms added); therefore, a two-stage minimization of the energy of the system was performed. Molecular-mechanical minimization was performed at the first stage to remove the largest strains in the system. Refinement of the active site structure was carried out at the second stage using the hybrid QM/MM method for energy minimization using the RM1 Hamiltonian. The stability of the resulting structure was confirmed by calculation of the 1,000 ps QM/MM MD trajectory. The calculated interatomic distances in the APE1 active site are listed in *Table* . It was demonstrated via the analysis of the resulting model that substrate binding in the active site of APE1 is accompanied by the formation of a number of bonds and interactions of a different nature. Among these, the hydrophobic interactions of deoxyribose of the AP site in the hydrophobic pocket formed by the Leu282, Phe266, and Trp280 residues should be noted. The free hydroxyl group in deoxyribose of the AP site also forms a hydrogen bond with the backbone carbonyl group of the Ala230 residue. The phosphate group at the 3’ terminus of the AP site is held by the positive charge of the Arg177 residue. The phosphate group under attack electrostatically interacts with the magnesium ion and forms hydrogen bonds with the side chains of Asn174, Asn212, and His309. The hydroxyl group of the Tyr171 residue is oriented towards the oxygen atom of the leaving group. 

**Fig. 2:**
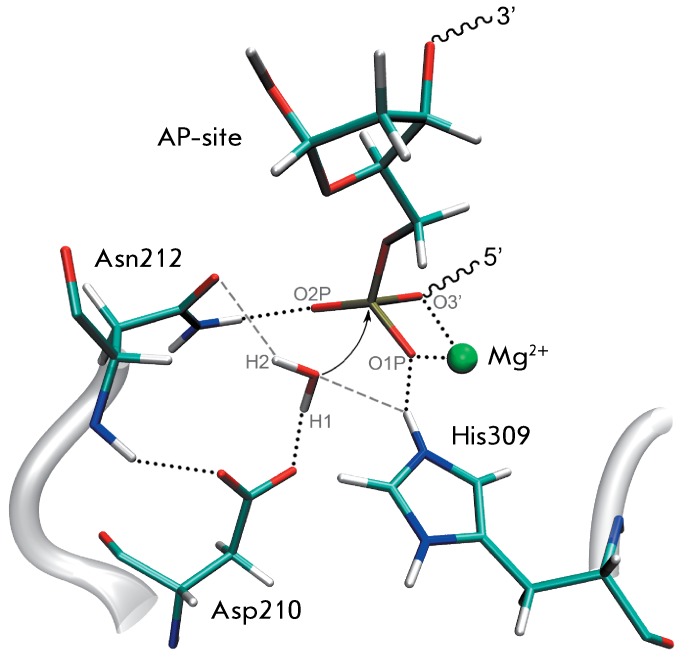
The active site of the full-atomic model of APE1 enzyme–substrate complex. Hydrogen bonds are shown as dotted lines. Electrostatic interactions other than hydrogen bonds are shown as dashed lines. The arrow shows the direction of nucleophilic attack.

The orientation of the attacking water molecule is provided via the interaction with the general base Asp210, carbonyl group of Asn212, and the side chain of His309; however, the interactions H _2_ O:H2 ··· Asn212:OD1 and H _2_ O:O ··· His309:HE2 are not conventional hydrogen bonds, since the average O···H2···OD1 and NE2···HE2···O angles are 137° and 122°, respectively. In the case of a hydrogen bond, these values should to be at least 150°. The reactive conformation of the carboxyl group of the general base Asp210 in the enzyme is maintained via the interaction between its side chain and the backbone amino group of Asn212 ( *Fig. 2* ). 

**Fig. 3:**
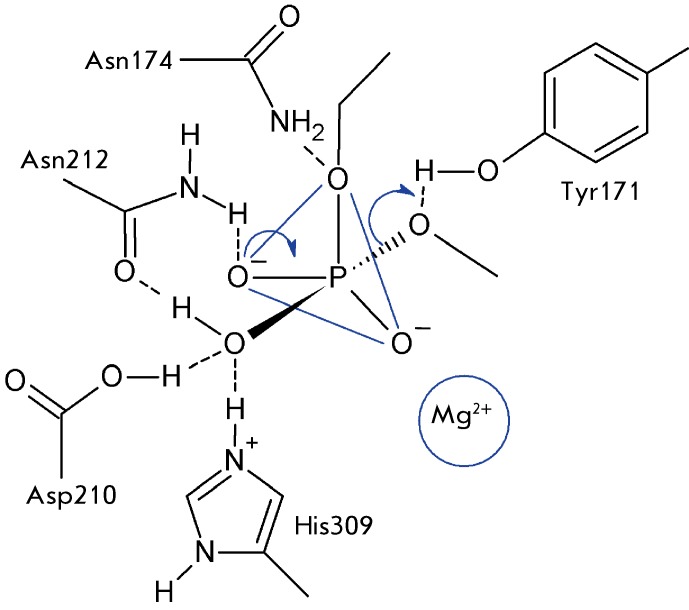
The schematic picture of the hypothetical structure of the transition state (intermediate shaped as a trigonal bipyramide) and its conversion upon a hydrolysis reaction catalyzed by endonuclease APE1.

Oriented and polarized by Asp210, His309, and the metal ion, the water molecule can attack the phosphate group of the substrate with the simultaneous transmission of the proton to the general base, the Asp210 residue. The intermediate resulting from the attack is shaped as a trigonal bipyramid and stabilized via the following interactions in the enzyme active site: oxygen atoms in the “apexes” of the bipyramid interact with the side chains of the His309 and Tyr171 residues; the trigonal base of the bipyramid is placed between Asn174, Asn212, and the magnesium ion ( *Fig. 3* ). 

The data pertaining to the mutagenesis on Tyr171 [15] attest to the crucial role of this residue in the catalytic mechanism of APE1; however, in contrast to the previous suggestions [15, 16], we assume a different role for this residue in the catalysis. The proximate location of the positive charge of magnesium ion and the Arg156 residue are supposed to facilitate proton migration from the hydroxyl group of Tyr171. Therefore, we consider this residue to be a potential proton donor for the leaving group, which is a strong base. The weaker influence of the mutations at position 171 on substrate binding in comparison with the decrease in the catalytic constant [15] is attributed to the weak interaction between the residue and the substrate at the earlier stages of the reaction preceding the catalytic process, which fully matches the assumption made. In the catalytic transformation, the leaving group apparently approaches the chain of the Tyr171 residue, enabling proton transfer. 

As the reaction proceeds, the less stabilized P–O ^-^ bond located at the base of the bipyramid and directed towards the Asn212 residue is subsequently transformed into a double P=O bond. The P–O bond directed towards the Tyr171 residue is simultaneously broken; as a result, the leaving group takes the proton away from the hydroxyl group of tyrosine ( *Fig. 3* ). 

Restoration of the catalytically active site (deprotonation of the general base Asp210 and protonation of the acid Tyr171) occurs via the interaction with surrounding water molecules. 

### Analysis of the binding of substrate and mechanism-dependent inhibitors 

A large number of charges and polar groups participate in the substrate binding and intermediate stabilization during the reaction. The character of these interactions and the ionization states of the amino acid residues of the active site define certain requirements to the structure of the substances capable of binding to the active site of APE1. It is necessary to implement the most significant interactions when constructing efficient inhibitors of the enzyme. The existence of the hydrophobic binding site, along with the number of polar groups and groups with various charges, further complicates the search for low-molecular-weight compounds of appropriate structure. Amino acids are a class of natural compounds the structure of which simultaneously contains substituents of a different nature, capable of both performing the hydrophobic and electrostatic interactions and of acting as hydrogen bond donor or acceptor. It has been noted [17] that 6-hydroxy-DOPA is a potential inhibitor of APE1endonuclease activity; however, an incorrect allowance was made for the ionization state of the inhibitor when modeling the enzyme–inhibitor interaction, making it more difficult to interpret the results. To clarify enzyme interactions with the potential inhibitors of this structure, molecular docking of different amino acids and their derivatives into the APE1 active site was carried out, taking into account the ionization states of the inhibitors and active site residues. 

**Fig. 4:**
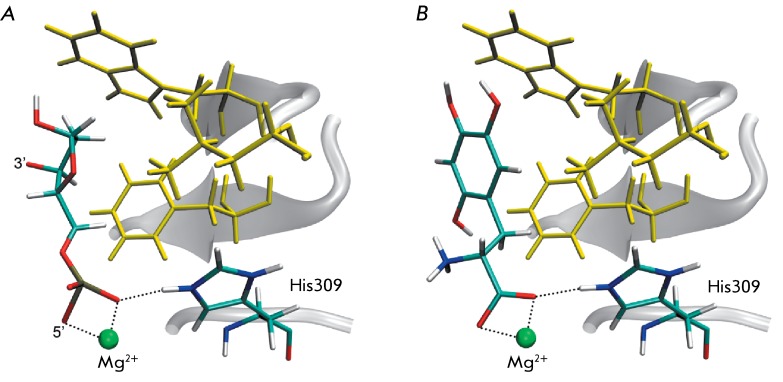
Positions of the substrate (A) and the potential inhibitor (B) in the active site of the full-atomic APE1 model. Residues of the hydrophobic pocket are shown in yellow: Phe266, Trp280, Leu282.

It was demonstrated via the analysis of the molecular docking results that the presence of the carboxyl group allows the selected compounds to bind to the metal ion and the His309 residue, whereas the hydrophobic substituent (e.g., phenyl radical) can occupy the hydrophobic pocket ( *Fig. 4* ). The introduction of hydroxyl substituents to the phenyl radical may lead to the formation of additional hydrogen bonds with the polar residues of the enzyme active site. Furthermore, the binding of the inhibitor to the charged residue of the general base is one of the factors presumably determining the inhibition efficiency. 

## CONCLUSIONS 

The aim of this work was to select the most reliable mechanism of action of APE1 on the basis of a molecular modeling, analysis of the full-atomic model of APE1, and a critique of the experimental results and assumptions previously made in the literature. For this purpose, calculation of the ionization states of the active site residues and a hybrid QM/MM modeling of the enzyme–substrate complex containing a water molecule capable of attacking the substrate were carried out. As a result of the investigation conducted, it was demonstrated that the Asp210 is likely to act as the general base in the catalytic mechanism, whereas the His309 residue, being protonated (and positively charged), participates in the binding of the phosphate group of the substrate. The analysis of the molecular dynamic trajectory of the enzyme–substrate complex attested to its high reactivity and confirmed the validity of the molecular modeling performed. 

The most important interactions in the active site determining the efficiency of binding of the substrate and the potential enzyme inhibitors (which are promising co-drugs of interest in the chemo- and radiotherapy of oncological diseases) were revealed. An assumption regarding the role of the Tyr171 of the active site of APE1 as the residue capable of ceding the proton to the leaving group of the substrate was made. Thus, the investigation enabled to establish a consistent mechanism of action of the enzyme. Furthermore, it allowed to summarize MD data, as well as the experimental results of kinetic studies and the other published data. In the next step, we plan to use higher level QM/MM methods to calculate the energy barrier of the reaction catalyzed by endonuclease APE1, in compliance with the mechanism proposed, and to screen for effective inhibitors with the use of the constructed mechanistic full-atomic model of the enzyme.  

## Abbreviations

BER: base excision repair
AP: apurinic/apyrimidinic
APE1: human apurinic/apyrimidinic endonuclease 1
QM/MM: quantum mechanics/molecular mechanics
MD: molecular dynamics
